# *Cryptosporidium* Genomics — Current Understanding, Advances, and Applications

**DOI:** 10.1007/s40475-024-00318-y

**Published:** 2024-03-23

**Authors:** Fiifi Agyabeng-Dadzie, Rui Xiao, Jessica C. Kissinger

**Affiliations:** 1 Department of Genetics, University of Georgia, Athens, GA 30602 USA; 2 Institute of Bioinformatics, University of Georgia, Athens, GA 30602 USA; 3 Center for Tropical and Emerging Global Diseases, University of Georgia, Coverdell Center, 107, 500 D.W. Brooks Drive, Athens, GA 30602 USA

**Keywords:** *Cryptosporidium*, Telomere-to-telomere, T2T, Single-oocyst sequencing, Hybrid capture, Transcriptomics, Population genomics

## Abstract

**Purpose of Review:**

Here we highlight the significant contribution that genomics-based approaches have had on the field of *Cryptosporidium* research and the insights these approaches have generated into *Cryptosporidium* biology and transmission.

**Recent Findings:**

There are advances in genomics, genetic manipulation, gene expression, and single-cell technologies. New and better genome sequences have revealed variable sub-telomeric gene families and genes under selection. RNA expression data now include single-cell and post-infection time points. These data have provided insights into the *Cryptosporidium* life cycle and host–pathogen interactions. Antisense and ncRNA transcripts are abundant. The critical role of the dsRNA virus is becoming apparent.

**Summary:**

The community’s ability to identify genomic targets in the abundant, yet still lacking, collection of genomic data, combined with their increased ability to assess function via gene knock-out, is revolutionizing the field. Advances in the detection of virulence genes, surveillance, population genomics, recombination studies, and epigenetics are upon us.

## Introduction

Cryptosporidiosis is a neglected disease, caused by apicomplexan parasites in the genus *Cryptosporidium*. It has devastating impacts on the most vulnerable, especially infants and the immunosuppressed [1]. As the scope and significance of this infectious disease has become apparent [2, 3••], the research community has responded. Over the last decade, there have been significant advances in genetics, genomics, the ability to culture the parasite and perform high-throughput screening, host–pathogen interactions, surveillance, and therapeutics. The advances in these areas have yielded large quantities of associated genomic data (Table 1) that are fueling advances in our understanding of *Cryptosporidium*, its life cycle [4, 5], its evolution [6], transmission [7••], and host–pathogen interactions [8, 9, 10••]. These data will also facilitate the design of better surveillance tools for local, regional, and hopefully, global use.

**Table 1:** Currently available *Cryptosporidium* genomic and transcriptomic data^#^

Species	Strain/isolate	Approach	Reference genome GenBank ID	Genome sequences	Assembly Status	Size range (Mb)	Genome sequences annotated	SRA gDNA data sets	SRA RNA data sets
*C. andersoni*	37,034 30,847 31,729	Illumina	GCA_001865355.1	3	Scaffold	8.966–9.089	1	5	0
*C. baileyi*	TAMU-09Q1	Illumina	GCA_001593455.1	1	Contig	8.494	0	5	1
*C. bovis*	45,015 SCAU24463 SCAU42270 SCAU24365 SCAU42290 SCAU24174 42,482	Illumina	GCA_009768925.2	7	Scaffold Contig	9.021–9.111	1	7	0
*C. canis*	33,844 45,460 25,894	Illumina	GCA_027243985.1	3	Scaffold Contig	8.553–8.746	3	4	0
*C. cuniculus*	UKCU2	Illumina	GCA_004337835.1	1	Scaffold	9.184	0	3	0
*C. felis*	44,884	Illumina	GCA_014529505.1	1	Scaffold	8.551	1	6	0
*C. hominis*	TU502 IbA9G3 TKD180701022-N703-AK415 TKD180701022-AK1581-AK428 37,999 30,976 TU502_2012 UKH1 UKH3 30,974 UKH5 UKH4 33,537 TU502	Illumina Ion torrent	GCA_000006425.1	15	Chr Scaffold Contig	8.692–9.180	5	391	4
*C. meleagridis*	UKMEL1 UKMEL3 UKMEL4 TU1867	Illumina ONT	GCA_001593445.1	3	Scaffold Contig	8.707–9.171	1	8	0
*C. muris*	RN66	Hybrid sequencing Sanger	GCA_000006515.1	1	Scaffold	9.245	1	5	0
*C. parvum*	IOWA II IOWA-ATCC IOWA-BGF IOWA UKP1 TU114 UKP6 HLJ 11730 Waterborne O18 UKP2 HB 12536 HN O20 GD 22971 UKP4 IIdA19G1 UKP3 UKP8 UKP7 UKP5 UKP16 UKP15 IIdA20G1 UKP14 IaA15G1R1 UKP12 UKP13 Iowa	Sanger Illumina Ion torrent ONT PacBio	GCA_000165345.1	27	Complete T2T Chr Scaffold Contig	8.882–9.402	4	698	145
*C. ryanae*	45,019	Illumina	GCA_009792415.2	1	Scaffold	9.059	1	1	0
*C. tyzzeri*	UGA55	Illumina	GCA_007210665.1	1	Scaffold	9.016	1	4	0
*C. ubiquitum*	39,726 39,668 39,725 UKUB2 UKUB1	Illumina	GCA_001865345.1	5	Scaffold	8.966–9.101	1	4	0
*C. viatorum*	UKVIA1	Illumina	GCA_004337795.1	1	Scaffold	9.264	0	1	0
Chipmunk genotype I	37,763	Illumina	GCA_004936735.2	2	Scaffold Contig	9.094–9.510	1	2	0
*Cryptosporidium* sp.	Chipmunk LX-2015	Illumina	GCA_000831705.1						
*Cryptosporidium* sp.*	SCAU42500	Illumina	GCA_029747635.1	1	Scaffold	9.094	1	1	0

*T2T* telomere to telomere

^#^Data were accessed at the NCBI GenBank in January 2024

*Unconfirmed *Cryptosporidium* species

Genomic data, however, are not without their challenges. Historically, *Cryptosporidium* genome sequences have been very hard to generate due to a lack of pure parasite material. This challenge has recently been overcome for genome sequence generation, but not for transcriptomics of post-infection *Cryptosporidium* life cycle stages. Another challenge arises from the fact that most available data were created with short-read sequencing approaches. While Illumina sequencing is highly accurate, it cannot yield complete genome assemblies and poses significant challenges for the analysis of gene families and repetitive sequences. Currently, cloning is also impossible for *Cryptosporidium*. Thus, nearly all genomic and transcriptomic data have been generated using populations of parasites rather than purified isogenic clones. This fact creates considerable challenges for genome assembly, data analysis, and interpretation. Here we highlight key advances, remaining challenges and future prospects.

## Technological Advances Have Facilitated *Cryptosporidium* Genomics

### Whole Genome Sequencing

With the advent of high accuracy second-generation sequencing (Illumina short reads) and the large fragment sequencing capabilities of third-generation sequencing (Pacific Biosciences and Oxford Nanopore Technologies, ONT), *Cryptosporidium* genome sequences are being generated at an increased rate [11]. Currently, there are 74 *Cryptosporidium* genome sequence assemblies located in the NCBI GenBank and more than half have been submitted since 2018. Whole-genome sequences are needed to facilitate the research community’s ability to design and interpret their experiments. As additional genome sequences become available for new species and strains, a framework for a more holistic genomic comparative analysis is being constructed. The power of comparative insights is significant [12, 13••, 14••, 15]. For example, the addition of a small number of genome sequences shed considerable insight into the diversity and evolution of a species, *C*. *parvum*, which revealed the existence of an anthroponotic subclade that was likely shaped via introgression of DNA from other *Cryptosporidium* species and subtypes [6].

### Single-Oocyst Sequencing

Genome sequence generation for *Cryptosporidium* has historically been quite difficult due to the large number of oocysts required for DNA preparation. Oocysts, which contain four haploid sporozoites, have ~ 40 fg of genomic DNA. Most clinical samples do not contain a sufficient number of oocysts to reach the minimum DNA requirements for sequencing library preparation. Thus, important isolates have historically been propagated in immunosuppressed mice or gnotobiotic pigs. This process is difficult, expensive, and time-consuming. However, with the advent of single-oocyst sequencing, the possibility of generating genomic sequences from a single oocyst is a reality [16]. The protocol involves oocyst sorting, lysis, genome amplification with multiple displacement amplification (MDA), and sequencing with short-read Illumina [16]. This technique has recently been modified to utilize long-read ONT sequencing [17••, 18]. Given the obligately sexual nature of *Cryptosporidium* and the existence of four related haploid sporozoites within an oocyst, single-oocyst sequencing is also a promising technique for studies of diversity within a single infection [16]. Single-oocyst sequencing also creates an avenue for studying diversity and recombination events within a single oocyst [17••].

### Hybrid Capture from Fecal DNA Samples

Hybrid capture, i.e., the selective enrichment of particular DNA sequences via hybridization to long, single-stranded RNA probes representing the target genome sequence of interest [19], is an ideal approach for isolating *Cryptosporidium* genomic DNA from fecal DNA samples. Most clinical *Cryptosporidium* fecal DNA samples contain abundant microbial, food, and human DNA content. Recently, hybrid capture has proven tractable for fecal DNA samples with a *Cryptosporidium* qPCR Ct score of < 20 and for much higher values if a double enrichment is performed (Bayona et al., in prep). The use of hybridization capture has made fecal DNA samples accessible for genome sequence generation. This development will permit samples from numerous studies, sitting in freezers to be analyzed. Importantly, the hybrid capture baits can be customized to have a wider sequence divergence range to facilitate detection of less common human-infecting species. Smaller subsets of probes can be tailored for specific regions of the genome to provide a multi-locus approach to quickly screen large numbers of samples and facilitate outbreak investigations.

### Genomic and Transcriptomic Data Are Abundant yet Incomplete

As we can see in Table 1, there are over 1000 genomic data sets for *C. parvum* and *C. hominis*; yet, there are only nine assembled and annotated genome sequences. Outside of these two prominent human-infecting species, the situation is bleak. A few dozen genome sequence data sets exist for all other species, and more than half of all named species have no genomic sequence data and are missing from Table 1.

**Fig. 1:**
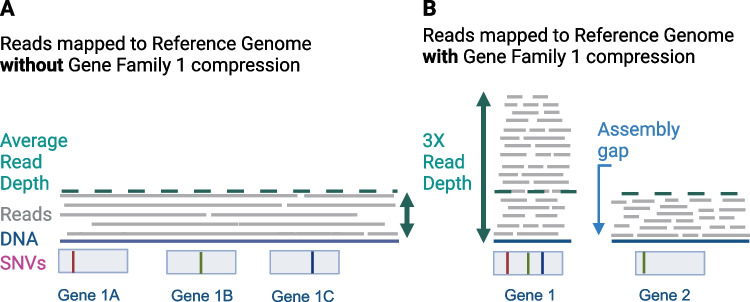
Genome assembly impacts annotation quality, gene family member estimates, and genetic variation analyses.** A** Long-read assembly can clearly identify all three copies of gene 1, and the average read depth is uniform suggesting the assembly does not contain compressed, i.e., merged, assembled sequence in this area. **B** Short-read assembly cannot separate the three closely related gene 1 family members as evidence by the read pile-up. The phenomenon is called a compression since three genes are merged and annotated as only 1 gene. Also, since the ends of the gene 1 reads are different for each gene copy, the contig cannot be extended, and a gap in the assembly is generated. Compressions do not only affect the determination of gene number; they also affect estimates of genetic variation. Reads that were generated from different family members are all mapped to one locus; thus, the estimate of variation is artificially high. This image was created with BioRender.com

Most existing *Cryptosporidium* genome sequences also present several challenges for the community. Most were generated using only short-read technologies that produce assemblies that contain gaps and compressed sequence regions (Fig. 1). A complete telomere-to-telomere, T2T, chromosomal assembly would contain eight chromosomes. Although the karyotype is unknown for most species, current assemblies contain dozens to hundreds of contigs, few telomere sequences, and many unassembled reads. Genome sequence assembly gaps most often arise in genome regions that contain repetitive sequences making short reads difficult to place. Long stretches of repetitive sequence also generate gaps, as does the merger of recent gene duplications that reside in multiple locations in genome (Fig. 1). Diversity within the population of parasites being sequenced can also create gaps because some parasites may possess structural variants like indels and inversions or differences in gene family or repeat copy numbers. When genome sequences contain gaps, it is difficult to know if genes are actually missing, thus posing significant challenges for comparative genomics.

The inclusion of long-read sequencing approaches and hybrid genome assemblies utilizing both long- and short-read approaches is the answer. Long reads, which can reach 100 + kb in length, can cover large genomic regions permitting an exact determination of repeat or gene copy numbers (Fig. 1) and provide proof of genome rearrangements. Depending on population numbers, contigs for differing genotypes within a population can be obtained, i.e., evidence for parasites that have two vs three copies of a particular gene in the same isolate.

When looking at RNA sequence data, the landscape is barren (Table 1), and most existing data sets are a combination of host and parasite transcripts since purification of post-infection parasites remains tenuous at best [20]. There are 145 RNA data sets for *C. parvum*, four for *C. hominis*, and one for *C. baileyi*. This paucity of data has significant consequences for the community. It means that the few genome sequences that have annotation have had to rely on orthology and de novo gene prediction alone. This means that species-specific genes are very difficult to discover, ncRNA genes will be missed, and untranslated regions (UTRs) will be unannotated making it difficult to know where promoters are located since transcription initiation sites are unknown and studies of post-transcriptional regulation, which often involve sequences in the 5′ and 3′ UTRs, are impossible.

## Comparative genomics of *Cryptosporidium* species yields informative insights

### Many Human-Infecting Species Are Closely Related

Genome sequences for the species most often observed in humans revealed that the genome sequences are highly similar and highly syntenic [6, 21, 22]. Several recent papers have also demonstrated the complex population genomic structure of *C. parvum* and *C. hominis* in natural infections [6, 7••, 15, 23] and highlighted the role that recombination and introgression have played during evolution [6, 14••, 15]. These and other works also show the impact that recombination can have with respect to the generation of novelty with proven impacts on transmission [7••]. Comparative genomics has also revealed the strikingly close relationship between the genome sequences of *C. cuniculus*, which infects rabbits and humans, and *C. hominis* [6]. Interestingly, an even closer genomic relationship was observed between the genome sequences of *C. parvum* and *C. tyzzeri*, yet *C. tyzzeri* only infects mice [24].

Recently, a genetic cross was reported not only within *C. parvum* but also between *C. parvum* and *C. tyzzeri* [17••]. In addition to being a major genetics breakthrough for *Cryptosporidium* research, this finding, combined with the recombination and introgression observations above, raises questions regarding the definition of what constitutes a species in *Cryptosporidium*. For many reasons, we are not advocating changes, only the recognition of just how similar some subclades and species are to each other at the genomic sequence level and how little we know about their host range [25]. *C. parvum* shares 96.8% identity with *C. hominis*, 97.2% identity with *C. tyzzeri*, 97% identity with *C. cuniculus*, and 91.3% identity with *C. meleagridis* (% identity is average nucleotide identity). These species also share almost complete synteny (gene order and orientation) and appear to differ by only a few sub-telomeric genes, if any [24, 26••] with the exception *of C. meleagridis* that appears, on the basis of long reads, to have a few dozen small intra- and inter-chromosomal rearrangements relative to the other species [27]. Thus, the genetic basis of host preference and pathogenicity may extend from gene content differences to also include single nucleotide variants, small indels, and possible differences in gene regulation. It is worth noting that significant differences in gene content between these species are found with short read analyses, including one in which some of us have participated [22, 28], highlighting the impact of technology and assembly quality on downstream analyses. Notably, synteny with species outside of this group, for which we have genome sequences, no longer extends for the full length of the chromosome and instead is broken down into smaller units of recognizable synteny [11, 29].

### Subtelomeric Chromosomal Regions Contain Gene Families and Appear to be Highly Dynamic

In general, the subtelomeric regions of eukaryotic chromosomes are more dynamic in terms of gene copy numbers and levels of observed variation than the rest of the chromosome, and this is particularly the case in pathogenic organisms [30, 31]. Genes that encode proteins involved in host–pathogen interactions and environmental responses are often, but not uniquely, located in sub-telomeric locations [30, 31]. As a result, these regions of the genome are notoriously difficult to assemble. They also represent some of the fastest evolving regions of the genome and, thus, are interesting from the perspectives of host–pathogen biology, evolution, and diagnostics/surveillance.

The first T2T genome sequences for *C. parvum* revealed surprises regarding higher than expected (based on previous short-read assemblies) gene copy number for a number of genes located in subtelomeric regions, e.g., MEDLE genes, tryptophan synthase beta, and rRNA genes among others [24, 26••]. They also revealed that three different chromosomes shared a total of four highly similar subtelomeric chromosome ends, indicating that replication had occurred between chromosomes [24, 26••]. Better assembly and identification of genes in subtelomeric regions are likely to be crucial for our understanding of important aspects of *Cryptosporidium* biology. For example, MEDLE proteins, most of which are encoded in subtelomeric regions, are important secreted pathogenesis determinants [9, 32, 33] that appear to be differentially present across a number of species [29].

### Population Genomic Studies Provide Insights into Variation, Evolution, and Transmission

Short-read sequencing technology permitted the generation of nearly 700 genomic data sets for *C. parvum* and nearly 400 for *C. hominis*. These highly accurate reads have been used to detect variants that exist among and between the different populations of *Cryptosporidium* parasites that have been sequenced [6, 7••, 13••, 14••, 15, 34, 35•]. The results have been illuminating. They have revealed a discordance in some cases between *gp60* single locus typing and genome ancestry, mixed infections with the same or different species, recombination events within species and hybridization between species, discovery of novel subclades, and in general demonstrated the role that admixture has had on shaping population structure [13••, 15].

These studies have also revealed how little we know about the global population structure of *Cryptosporidium* species and the forces driving their evolution in differing environments and outbreak scenarios [6, 7••, 13••, 14••, 15, 34, 35•]. These studies also reveal the critical role that the reference genome has in the determination of differences in gene content and polymorphisms. Figure 1 highlights the theoretical outcome of determining single-nucleotide variants (SNVs) in two different scenarios, uncompressed gap free and compressed gapped genome sequences. Thus, a degree of caution is warranted for the interpretation of variant calling until the community has more complete reference genome sequences. The community would greatly benefit from a more diverse set of reference genome sequences and methods for capturing novel genomic content that may not be present in any given reference genome sequence.

## Transcriptomics in *Cryptosporidium*

### Annotation, Antisense, and ncRNA Transcripts

Utilization of small RNA-seq and PacBio long-read Iso-seq and ONT Direct RNAseq has significantly advanced our understanding of the *Cryptosporidium* transcriptome. These technologies have enabled the identification of untranslated regions (UTRs), as well as a variety of long and short non-coding RNAs (ncRNAs), including anti-sense transcripts of unknown function [26••, 36, 37]. Furthermore, single-molecule long-read RNAseq has been instrumental in demonstrating that approximately 10% of *C. parvum* genes have polycistronic transcripts, offering new insights into gene expression biology and regulation in this important pathogen (Xiao et al., in prep).

### Differential Gene Expression

There is a significant amount of RNA-seq data for *C*. *parvum* (Table 1). Studies involving oxidative and heat stress on *C. parvum* oocysts have identified genes responsive to environmental cues [38]. Transcriptomic analyses of *C. parvum*-infected HCT-8 cells have revealed gene regulation patterns during early stages of infection [39•]. Comparative RNA-seq studies of both *C. parvum* and *C. hominis* have provided novel data sets on host-parasite RNA interactions during infection [40]. Research on AP-2 transcription factor deletion and its impact on sex differentiation and oocyst shedding, RNA m6A-immunoprecipitation in infected host cells, and differential gene expression in distinct *Cryptosporidium* species using enteroids have all contributed significantly to our understanding of life cycle progression and interactions with the host [41••, 42, 43••]. Additionally, RNA-seq in drug screening assays has suggested potential inhibition of translation during parasite sexual differentiation [44].

RNA expression data sets are desperately needed for additional species, especially *C. hominis* (Table 1). These data will permit comparative transcriptomic analyses and provide additional insight into gene regulatory differences that may exist between species. Additionally, RNA data can also be used to greatly improve the annotation of reference genome sequences for the community by providing evidence for UTRs and alternative isoforms, if present.

### Epigenetics

Recent studies have shown that *C. parvum* has enzymatically functional histone methyltransferases, indicating developmentally dependent histone modifications. These findings also suggest that *C. parvum* infection can alter the epigenetic landscapes of host cells [45••]. Additionally, ATAC-seq in *C. parvum* sporozoites has provided the first glimpse into the parasite’s accessible chromatin landscape, prior to invasion, enabling new research into the regulation of parasite gene expression (Xiao et al., in prep). The community will benefit greatly from additional research into this exciting layer of gene regulation in *Cryptosporidium* especially during it developmental life cycle.

## Major Advances That Are Generating Abundant, Informative Genomic and Transcriptomic Data

### Transgenics

CRISPR/Cas9 mediated genetic modifications work in *Cryptosporidium* [46, 47]. Genetically modified *C. tyzerri* and *C. parvum* are being used to study host protective immunity and *Cryptosporidium* biology [47–49]. Dihydrofolate reductase-thymidylate synthase (DHFR-TS) and inosine monophosphate dehydrogenase (IMPDH) have been knocked out to study nucleotide synthesis [50]. *C. parvum* parasites were still viable after gene knockouts, suggesting alternative pathways for sequestering nucleotides from the host. This knowledge will help in drug development as some drugs are designed to target nucleotide synthesis [50], and these pathways have been successfully targeted in other apicomplexans.

Several selectable genetic markers are now available [17••, 46, 51••], and this development has permitted genetic crosses [17••, 51••] in immunocompromised mice. This advance is of considerable significance. The model, combined with genomic sequences, opens up reverse genetic research into *Cryptosporidium* biology, development, and the important question of host specificity.

As cloning of individual parasites is not yet possible, many phenotypic effects like changes in gene regulation or recombinant progeny are assessed via analyses of genome and transcriptome sequencing [17••], thus generating many new, important, data sets.

### RNA Host–Pathogen Interactions

Recent advances looking at host-*Cryptosporidium* interactions have revealed that a wide variety of novel RNA forms are involved. For example, host circular RNA ciRS-7 is upregulated during *C. parvum* infection in HCT-8 cells. This upregulation influences the NF-κB signaling pathway by sponging miR-1270, which in turn significantly impacts the propagation of *C. parvum* [52]. A recent scRNA study of *C. parvum*-infected intestinal epithelial cells has led to development of a model to explain the role of IFN-gamma in the control of *C. parvum* infection in intestinal epithelial cells [53].

On the parasite side, recent research has shown that some *C. parvum* long noncoding RNAs can localize to the host cell [10••] and manipulate host cell gene expression by suppressing expression of CDH3 and LOXL4 [8]. One group has also explored RNA-based therapy, in which a single-stranded antisense RNA designed to parasite protein coding genes can silence parasite genes when loaded with argonaute protein [54].

### The *Cryptosporidium* RNA Virus and Host–Pathogen Interactions

Previous studies have discovered two unique extrachromosomal linear double-stranded RNAs in *C. parvum*, encoding an RNA-dependent RNA polymerase and a protein kinase [55] The *Cryptosporidium* dsRNA virus has been detected in isolates of *C. parvum*, *C. hominis*,* C*. *meleagridis*, and *C. felis* [56]. Recent research has shown that the dsRNA virus *can* hijack a host long noncoding RNA, U90926 [10••]. Additional research has also shown that the *Cryptosporidium* dsRNA virus can trigger the host type I IFN antiviral pathway to dampen the hosts antiparasitic response, thus facilitating parasite success [57••]. All of these studies have generated numerous, exciting, host–pathogen gene expression data sets.

## Applications to Control and Prevention

### The Influence of Genomics on *Cryptosporidium* Surveillance

The natural environments that serve as reservoirs for *Cryptosporidium* are waterbodies and hosts. Several detection techniques have been developed and improved significantly, from conventional microscopy to immunological assays, flow cytometry, and nucleic acid–based methods [58]. Nucleic acid–based detection methods are highly efficient in detecting mixed infections and mixed populations with low abundance subpopulations present [59]. With nucleic acid detection–based approaches such as PCR, qPCR, and DNA sequencing, tiny amounts of *Cryptosporidium* DNA can be detected, and new bioinformatics tools make it easier to type Sanger sequences of *Cryptosporidium s*pecies based on *gp60* and SSU rRNA [60]. Though DNA sequencing as a detection method is relatively expensive, the ability to multiplex samples makes it more affordable, and its high accuracy makes it a better choice than microscopy [61, 62]. Accurately detecting and identifying *Cryptosporidium* sp. in an outbreak, based on genomic data, facilitate inquiries into the source of the outbreak and, on a broader scale, the epidemiology of the disease [63].

Clinical samples are essential in the study of cryptosporidiosis. Unfortunately, clinical isolates do not contain enough oocysts for traditional sequencing as discussed above. However, multiple displacement amplification (MDA), a type of whole genome amplification (WGA) technique, can be used to amplify the amount of DNA. MDA introduces very few errors, amplification is random, and the entire genome can be amplified for sequencing [64, 65]. WGA provides a means to obtain sufficient DNA from samples for use in analyses as well as long-read sequencing [66]. Studies of parasite diversity within and between samples can inform upon the source of the infection and the diversity of *Cryptosporidium* circulating within the population.

### Genetic Markers, Diagnostics, and Surveillance

Phenotypic and antibody diagnostic identification are unable to distinguish *Cryptosporidium* species and subtypes; therefore, the genetic markers used such as 18SrRNA and *gp60* were developed as useful single genetic markers. Due to the sexual nature of *Cryptosporidium*, and the fact that it has eight independently segregating chromosomes, multi-locus typing is a more ideal approach to typing [67]. Although there is no consensus on the specific markers to use, there is agreement that a multi-locus approach is needed [68] to better inform on *Cryptosporidium* epidemiology. Also, multi-locus genotyping is better because more data are available for identification and it adds the possibility of species subtype identification [69].

The increasing availability of full genome sequence data from increasing numbers of isolates should make the determination of appropriate loci for typing easier, but challenges remain. First, the community still lacks genomic sequence data from isolates circulating in many important regions of the world with a high incidence of *Cryptosporidium* infection. Just compare the burden reported in Gilbert et al. [3••] with the source of available genomes sequences in Fan et al. [11]. This situation is beginning to change, and sequences from isolates in other countries are emerging [15]. Hopefully, the genomic advances described above will facilitate this process and unlock the potential of existing samples and lead to the strategic collection of others. Additional genome sequences from new geographic locations and environments will allow the community to survey the extent of the genomic diversity that exists globally and design markers to account for it. One can also imagine the need for specialized markers to very quickly evolving regions of the genome that can be utilized in outbreak scenarios to detect variants as they arise.

Second, in order to appropriately assess genomic variation and rapidly evolving genomic regions, complete, T2T reference genomes for the species most commonly infecting humans should be established and adopted.

Finally, markers for routine surveillance are also needed. RT-PCR tests for the *Cryptosporidium* dsRNA virus are very sensitive due to viral abundance [70]. The method has been used to successfully identify *Cryptosporidium* infection in calves, lambs, goats, and environmental water samples across the world [71–73]. However, we do not yet know the full extent to which the dsRNA virus is present in different species [56].

## Conclusions

Advances in genomics and transcriptomics are impacting all arenas of *Cryptosporidium* research [74••] from evolution to the life cycle, to host–pathogen interactions and surveillance. The *Cryptosporidium* research community has come far, very quickly with many new technologies, approaches, and data sets. Much of this new data is available for use and mining in the NCBI GenBank [75] and CryptoDB.org [76•].

The *Cryptosporidium* community is also struggling a bit with the difficult challenges posed by this important pathogen and the state of genomics technology. The lack of transcriptomic data for species other than *C. parvum* and the lack of genome sequences for more than half of the named species are real challenges. Complete genome sequences are still too hard to generate and even harder to consistently annotate, especially in the absence of RNA data. This reality impacts their utility and application to important needs like global surveillance and determination of complete gene repertoires. Complete, annotated reference genome sequences greatly facilitate experimental design, e.g., gene knock-outs, and pathway analyses. Likewise, analyses of data that require a reference genome sequence for interpretation, e.g., transcriptome and proteome data analyses and comparative genomics and evolution studies, will also benefit.

The community is also struggling with appropriate geographic representation of genomic data sets from many of the countries most affected by this pathogen. This lack of representation impacts the development of more representative, multi-locus diagnostics and impacts our knowledge base for epidemiological studies and outbreak investigations. Given how difficult *Cryptosporidium* is to work with, genomics advances have come far, but more is needed.

## Data Availability

No datasets were generated or analysed during the current study.

## Declarations

### Disclaimer

The funders played no role in the study design, data collection and analysis, decision to publish, or preparation of the manuscript.
